# Supplementary material for: Minimal Cycle Representatives in Persistent Homology Using Linear Programming: An Empirical Study With User’s Guide
Source: Front Artif Intell. 2021 Oct 11;4:681117. doi: 10.3389/frai.2021.681117 (PMC8544243; doi:10.3389/frai.2021.681117)
Supplement: Supplementary file 1 [file DataSheet1.pdf]

Supplementary Material

1 REVIEW: STANDARD ALGORITHM TO COMPUTE PERSISTENT HOMOLOGY CYCLE BASES, BY MATRIX DECOMPOSITION

The first step in Algorithms 1 and 2 is to compute a persistent homology cycle basis. The standard method to compute such a basis invokes a so-called $R = DV$ decomposition of the boundary matrices ∂_n Cohen-Steiner et al. [2006]. Here we provide a brief review of this process; further details may be found in Cohen-Steiner et al. [2006]; de Silva et al. [2011].

To begin, we must place a total order on each set $\mathbf{S}_n(K)$, in ascending order of birth. This naturally allows us to regard ∂_n as an element of $G^{|\mathbf{S}_{n-1}(K)| \times |\mathbf{S}_n(K)|}$. The *low* function on a matrix $A \in G^{k \times l}$ is defined by

$$\text{low} : \underbrace{\{j : A[:, j] \neq 0\}}_{\text{domain(low)}} \to \mathbb{Z}, \quad j \mapsto \max\{i : A[i, j] \neq 0\}.$$

We say that A is *reduced* if low is injective. An $R = DV$ decomposition is a matrix equation where R is reduced and V is invertible and upper triangular.

Suppose that $R_n = \partial_n V_n$ is such a decomposition for each n. Let low^n be the low function of R_n, and let $\Gamma_n = \{(j, \text{low}(j)) : R[:, j] \neq 0\}$ be the graph low^n. It can then be shown (Cohen-Steiner et al. [2006]; de Silva et al. [2011]) that

1. Each set $\mathbf{S}_n(K)$ partitions into three disjoint subsets: $B_n \sqcup B_n^* \sqcup H_n$, where B_n is the image of low^{n+1} and B_n^* is the domain of low^n.
2. If $B_n^*(t)$ denotes the set of simplices in B_n born by time t, then $\partial_n[:, B_n^*(t)]$ is a basis for the space of boundaries $\mathbf{B}_n(K_t)$.
3. Let $\hat{\Gamma}_n$ denote the subset of Γ_n consisting of those pairs (σ, τ) such that $\text{Birth}(\sigma) \neq \text{Birth}(\tau)$, and let $E_n = \{\tau : (\sigma, \tau) \in \hat{\Gamma}\} \cup H_n$. Then $V_n[:, H_n] \cup R_n[:, E_n]$ is a persistent homology cycle basis. The lifespan of $V_n[:, \sigma]$ is $[\text{Birth}(\sigma), \infty)$ and the lifespan of $R_n[:, \tau]$ is $[\text{Birth}(\sigma), \text{Birth}(\tau))$, where σ is the unique simplex such that $(\sigma, \tau) \in \Gamma$.
4. In particular, the barcode of $K_\bullet$ may be read off from the $R = DV$ decompositions.

2 CORRECTNESS OF ALGORITHMS 1 AND 2

Here we provide proofs of correctness for Algorithms 1 and 2. As the details are primarily technical in nature, the exposition is fairly terse. The arguments are primarily self-contained, however we begin by recalling one result from Henselman and Ghrist [2016], which will be used in the proof of Algorithm 1.

2.1 Review: characterization of persistent homology bases

We will invoke a result from Henselman and Ghrist [2016] and Henselman-Petrusek [2017] which requires one new definition and one new notational convention. For notation, let n be given, fix $\epsilon_i < \epsilon_j$, and set

$$X^{i,j} := \mathbf{Z}_n(K_{\epsilon_i}) \cap \mathbf{B}_n(K_{\epsilon_j}) \qquad Y^{i,j} := \mathbf{Z}_n(K_{\epsilon_{i-1}}) + \mathbf{B}_n(K_{\epsilon_{j-1}}) \qquad Q^{i,j} := \frac{X^{i,j}}{X^{i,j} \cap Y^{i,j}}$$

We then define $q^{i,j}$ as the quotient map

$$q^{i,j} : X^{i,j} \to Q^{i,j}$$

Definition 2.1. A subset $E \subseteq X^{i,j}$ is an (i,j)-*basis* if $q^{i,j}|_E$ is injective and $q^{i,j}(E)$ is a basis of $Q^{i,j}$.

THEOREM 2.2 (Henselman and Ghrist [2016]; Henselman-Petrusek [2017]). *A family of n-cycles E is a persistent homology cycle basis iff*

$$E^{i,j} := E \cap (X^{i,j} - Y^{i,j})$$

is an (i,j) basis for all i and j.

Remark 2.3. A special case of Theorem 2.2 (for simplex-wise filtrations) also appeared in [Wu et al., 2017, Theorem 1]. This construction is also closely related to that of *pair groups*, c.f. Edelsbrunner and Harer [2008].

2.2 Correctness of Algorithm 1

We restate Algorithm 1 for ease of reference.

Algorithm 1 Edge-loss persistent cycle minimization

Compute a persistent homology basis $\mathcal{B}$ for homology in dimension 1, with coefficients in $\mathbb{Q}$, using the standard matrix decomposition procedure described in the Supplementary Material. Arrange the elements of $\mathcal{B}$ into an ordered sequence $Z^0 = (\mathbf{z}^{0,1}, \ldots, \mathbf{z}^{0,m})$.
for $j = 0, \ldots, m-1$ **do**
  Solve Program (14) to optimize the $j+1$th element of Z^j. Let $\mathbf{x}$ denote the solution to this problem, and define Z^{j+1} by replacing the $j+1$th element of Z^j with $\mathbf{x}$. Concretely, $\mathbf{z}^{j+1,j+1} = \mathbf{x}$, and $\mathbf{z}^{j+1,k} = \mathbf{z}^{j,k}$ for $k \neq j$.
end for
Return $\mathcal{B}^* := \{\mathbf{z}^{m,1}, \ldots, \mathbf{z}^{m,m}\}$, the set of elements in Z^m.

Recall that Program (14) optimizes the jth element of an ordered sequence of cycle representatives $Z = (\mathbf{z}^1, \ldots, \mathbf{z}^m)$. In particular, it seeks to minimize $\mathbf{x}^{Orig} := \mathbf{z}^j$. To define this program, we first construct a matrix A such that $A[:, i] = \mathbf{z}^i$ for $i = 1, \ldots, m$. We then define three index sets, $\mathcal{P}, \mathcal{Q}, \mathcal{R}$ such that

$$\mathcal{P} = \{i : \text{Birth}(\mathbf{z}^i) \leq \text{Birth}(\mathbf{x}^{Orig}),\ \text{Death}(\mathbf{z}^i) \leq \text{Death}(\mathbf{x}^{Orig}),\ i \neq j\}$$
$$\mathcal{Q} = \{\sigma \in \mathbf{S}_2(K) : \text{Birth}(\sigma) \leq \text{Birth}(\mathbf{x}^{Orig})\}$$
$$\mathcal{R} = \{\sigma \in \mathbf{S}_1(K) : \text{Birth}(\sigma) \leq \text{Birth}(\mathbf{x}^{Orig})\}$$

Program (14) can then be defined as follows.

$$
\begin{aligned}
&\text{minimize } ||W\mathbf{x}||_1 = \sum_{i=1}^{N}(x_i^+ + x_i^-)\\
&\text{subject to } (\mathbf{x}^+ - \mathbf{x}^-) = \mathbf{x}^{Orig}[\mathcal{R}] + \partial_2[\mathcal{R},\mathcal{Q}]\mathbf{q} + A[\mathcal{R},\mathcal{P}]\mathbf{p}\\
&\qquad\qquad \mathbf{p} \in \mathbb{Q}^{\mathcal{P}}\\
&\qquad\qquad \mathbf{q} \in \mathbb{Q}^{\mathcal{Q}}\\
&\qquad\qquad \mathbf{x} \in G^{\mathcal{R}}\\
&\qquad\qquad \mathbf{x}^+, \mathbf{x}^- \geq 0
\end{aligned}
\tag{14}
$$

THEOREM 2.4. *For each k, the family of cycles $\{\mathbf{z}^{k,1}, \dots, \mathbf{z}^{k,m}\}$ constructed in Algorithm 1 is a persistent homology cycle basis. Moreover, lifespans are preserved, in the sense that*

$$\mathcal{L}(\mathbf{z}^{0,l}) = \mathcal{L}(\mathbf{z}^{k,l}) \tag{S1}$$

for all k and l.

PROOF. We proceed by induction on k, the base case $k = 0$ begin clear. Assume the desired conclusion holds for k. For ease of reference, put

$$Z := (\mathbf{z}^1, \dots, \mathbf{z}^m) := (\mathbf{z}^{k,1}, \dots, \mathbf{z}^{k,m}) = Z^k. \qquad \mathbf{x}^{Orig} := \mathbf{z}^{k+1} \qquad [\epsilon_i, \epsilon_j) := \mathcal{L}(\mathbf{x}^{Orig})$$

We may then partition $\mathcal{P}$ as the disjoint union $\mathcal{S} \sqcup \mathcal{T}$, where

$$
\begin{aligned}
\mathcal{S} &= \{l \in \mathcal{P} : \text{Birth}(\mathbf{z}^l) < \text{Birth}(\mathbf{z}^{k+1}) \text{ or } \text{Death}(\mathbf{z}^l) < \text{Death}(\mathbf{z}^{k+1})\}\\
\mathcal{T} &= \{l \in \mathcal{P} : \text{Birth}(\mathbf{z}^l) = \text{Birth}(\mathbf{z}^{k+1}),\ \text{Death}(\mathbf{z}^l) = \text{Death}(\mathbf{z}^{k+1}),\ l \neq k+1\}.
\end{aligned}
$$

An optimal solution to Program (14) can then be expressed in form

$$\mathbf{x} = \mathbf{z}^{k+1} + \partial_2[\mathcal{R},\mathcal{Q}]\mathbf{q} + A[\mathcal{R},\mathcal{P}]\mathbf{q} = \mathbf{z}^{k+1} + \underbrace{\partial_2[\mathcal{R},\mathcal{Q}]\mathbf{q}}_{\mathbf{u}} + \underbrace{A[\mathcal{R},\mathcal{S}]\mathbf{q}[\mathcal{S}]}_{\mathbf{v}} + \underbrace{A[\mathcal{R},\mathcal{T}]\mathbf{q}[\mathcal{T}]}_{\mathbf{w}}$$

where

$$\mathbf{u} \in \mathbf{B}(K_{\epsilon_i}) \subseteq X^{i,j} \cap Y^{i,j} \qquad \mathbf{v} \in X^{i,j} \cap Y^{i,j} \qquad \mathbf{w} \in \text{span}(\{\mathbf{z}^t : t \in \mathcal{T}\}).$$

Now put

$$
\begin{aligned}
F &:= \{\mathbf{z}^t : t \in \mathcal{T}\} \sqcup \{\mathbf{z}^{k+1}\}\\
F' &:= \{\mathbf{z}^t : t \in \mathcal{T}\} \sqcup \{\mathbf{z}^{k+1} + \mathbf{w}\}\\
F'' &:= \{\mathbf{z}^t : t \in \mathcal{T}\} \sqcup \{\mathbf{z}^{k+1} + \mathbf{u} + \mathbf{v} + \mathbf{w}\} = \{\mathbf{z}^t : t \in \mathcal{T}\} \sqcup \{\mathbf{x}\}
\end{aligned}
$$

Since $\mathbf{w} \in \mathrm{span}(\{\mathbf{z}^t : t \in \mathcal{T}\})$, it is easily argued that $\mathrm{span}(F) = \mathrm{span}(F')$. Thus $\mathrm{span}(q^{i,j}(F)) = \mathrm{span}(q^{i,j}(F')) = \mathrm{span}(q^{i,j}(F'')) = Q^{i,j}$. Dimension counting thus implies that $q^{i,j}(F'')$ is an (i,j)-basis.

Given this observation, it is straightforward to verify that $\{\mathbf{z}^{k+1,1}, \ldots, \mathbf{z}^{k+1,m}\}$ is a bona-fide cycle basis and $\mathcal{L}(\mathbf{x}) = \mathcal{L}(\mathbf{x}^{Orig})$. The desired conclusion follows.

This establishes our primary objective:

THEOREM 2.5. *The set $\mathcal{B}^*$ returned by Algorithm 1 is a bona fide persistent homology cycle basis of the filtered simpicial complex $K_\bullet$.*

2.3 Correctness of Algorithm 2

Recall that Obayashi [2018] defines a *persistent volume* for a birth-death pair $(\sigma_{b_i}, \sigma_{d_i})$ as an $(n+1)$ chain $\mathbf{v} \in \mathbf{C}_{n+1}(K_{d_i})$ such that

$$\mathbf{v} = \sigma_{d_i} + \sum_{\sigma_k \in \mathcal{F}_{n+1}} \alpha_k \sigma_k \tag{S2}$$

$$(\partial_{n+1}\mathbf{v})_\tau = 0 \quad \forall \tau \in \mathcal{F}_n \tag{S3}$$

$$(\partial_{n+1}\mathbf{v})_{\sigma_{b_i}} \neq 0, \tag{S4}$$

where

$$\mathcal{F}_l := \{\sigma_k \in \mathbf{S}_l(K) : b_i < k < d_i\} \tag{S5}$$

is the family of l-simplices whose birth time lies strictly between b_i and d_i. The linear program associated to $(\sigma_{b_i}, \sigma_{d_i})$ in Obayashi [2018] can then be summarized as

$$\begin{aligned} &\text{minimize } \mathrm{loss}(\mathbf{v}) \\ &\text{subject to (S2), (S3), (S4)} \\ &\qquad\quad \mathbf{v} \in \mathbf{C}_{n+1}(K_{d_i}) \end{aligned} \tag{10}$$

Let us restate Algorithm 2 and the corresponding optimization problem, Program (15), for ease of reference.

Recall that we refer to Program (15) as the *general triangle-loss problem.*

$$\begin{aligned} \text{minimize } & ||W\mathbf{v}||_1 = \sum_{i=1}^{N}(v_i^+ + v_i^-) \\ \text{subject to } & \partial_{n+1}[\sigma, \hat{\mathcal{F}}_{n+1}]\mathbf{v} \neq 0 \\ & \partial_{n+1}[\mathcal{F}_n, \hat{\mathcal{F}}_{n+1}]\mathbf{v} = 0 \\ & \mathbf{v}_\tau = 1 \\ & \mathbf{v}^+, \mathbf{v}^- \geq 0 \\ & \mathbf{v}^+, \mathbf{v}^- \in G^{\hat{\mathcal{F}}_{n+1}} \end{aligned} \tag{15}$$

Algorithm 2 Triangle-loss persistent cycle minimization

1. Place a filtration-preserving linear order $\leq^{(l)}$ on $\mathbf{S}_l(K)$ for each l.
2. Compute an $R = \partial_{n+1}V$ decomposition as described in Cohen-Steiner et al. [2006] and the Supplementary Material. We then obtain a set Γ of birth/death pairs (σ, τ).
3. For each $(\sigma, \tau) \in \Gamma$ such that $\mathrm{Birth}(\sigma) < \mathrm{Birth}(\tau)$, put
$$\mathcal{F}_n := \{\sigma' \in \mathbf{S}_n(K) : \mathrm{Birth}(\sigma') \leq \mathrm{Birth}(\tau),\ \sigma \lneq^{(n)} \sigma'\}$$
$$\mathcal{F}_{n+1} := \{\tau' \in \mathbf{S}_{n+1}(K) : \mathrm{Birth}(\sigma) \leq \mathrm{Birth}(\tau'),\ \tau' \lneq^{(n+1)} \tau\}$$
and $\hat{\mathcal{F}}_{n+1} := \mathcal{F}_{n+1} \cup \{\tau\}$. Compute a solution to the corresponding Program (15), and denote this solution by $\mathbf{x}^{\sigma,\tau}$.
4. Put $\hat{\mathcal{D}} := \{\partial_{n+1}(\mathbf{x}^{\sigma,\tau}) : (\sigma, \tau) \in \Gamma \text{ and } \mathrm{Birth}(\sigma) < \mathrm{Birth}(\tau)\}$ and let $\hat{\mathcal{D}}' := \{\mathbf{z} \in \mathcal{M} : \mathrm{Death}(\mathbf{z}) = \infty\}$, where $\mathcal{M}$ is a persistent homology cycle basis calculated by the standard $R = DV$ method.
5. Return $\mathcal{D} := \hat{\mathcal{D}} \cup \hat{\mathcal{D}}'$.

To verify that Algorithm 2 returns a bona-fide persistent homology cycle basis, let us begin by placing the elements of K into a sequence $(\sigma_1, \ldots, \sigma_{|K|})$ by ordering simplices first by birth time, second (that is, breaking ties when birth times agree) by dimension, and finally (breaking ties when birth times and dimensions agree) by the chosen linear orders $\leq^{(l)}$. It is simple to verify that this rule defines a unique linear order on K, and that the filtration $K'_\bullet$ defined by

$$K'_i := \{\sigma_1, \ldots, \sigma_i\}$$

is a simplex-wise refinement of $K_\bullet$.

THEOREM 2.6. *Let (σ, τ) be a birth-death pair, and choose b_i, d_i such that $(\sigma_{b_i}, \sigma_{d_i}) = (\sigma, \tau)$. Then the sets $\mathcal{F}_n$ and $\mathcal{F}_{n+1}$ defined in Algorithm 2 both satisfy* (S5). *Consequently, Program* (15) *is a special case of Program* (10).

PROOF. The proof is a straightforward exercise in definition checking.

Now let $\hat{\mathcal{B}}$ be a set containing the boundary of one optimal solution $\mathbf{x}^{\sigma,\tau}$ to Program (15) for each birth-death pair $(\sigma, \tau) = (\sigma_{b_i}, \sigma_{d_i})$ (even if $\mathrm{Birth}(\sigma) = \mathrm{Birth}(\tau)$)). Let $\mathcal{N}$ be a persistent homology cycle basis for $K'_\bullet$, and let $\hat{\mathcal{B}}' = \{\mathbf{z} \in \mathcal{N} : \mathrm{Death}(\mathbf{z}) = \infty\}$ be the collection of cycle representatives in $\mathcal{N}$ that never die. Then, by combining Theorem 2.6 with Theorem 5 of Obayashi [2018], we find that $\mathcal{B} := \hat{\mathcal{B}} \cup \hat{\mathcal{B}}'$ is a persistent homology cycle basis of $K'_\bullet$.

If we assume that the bounding volumes used to obtain $\hat{\mathcal{B}}$ are the same as those used to obtain $\hat{\mathcal{D}}$ in Algorithm 2, and, likewise, that $\hat{\mathcal{B}}' = \hat{\mathcal{D}}'$ (this will be true provided we use the order $\leq^{(l)}$ when ordering columns of ∂_l for the $K_\bullet$ calculation) then

$$\mathcal{D} = \{\mathbf{z} \in \mathcal{B} : \mathrm{Birth}(\mathbf{z}) < \mathrm{Death}(\mathbf{z})\}$$

where Birth and Death are the birth and death functions of $K_\bullet$, not $K'_\bullet$.

From here, it remains only to verify that $\mathcal{D}$ is a bona fide persistent homology cycle basis $K_\bullet$. This follows from the following general observation.

THEOREM 2.7. *Let $L'_\bullet$ be a refinement of a filtration $L_\bullet$ on a simplicial complex L, and let $\mathcal{B}$ be a persistent homology cycle bases for $L'_\bullet$. If* Birth *and* Death *are the birth and death functions of $L_\bullet$ and $\mathcal{D} := \{\mathbf{z} \in \mathcal{B} : \mathrm{Birth}(\mathbf{z}) < \mathrm{Death}(\mathbf{z})\}$, then $\mathcal{D}$ is a persistent homology cycle basis of $L_\bullet$.*

PROOF. Recall that, by definition, a set $\mathcal{E}$ is a persistent homology cycle basis of $K_\bullet$ iff two criteria hold: (i) $\mathcal{L}(\mathbf{z})$ must be nonempty for each $\mathbf{z} \in \mathcal{E}$, and (ii) $\{[\mathbf{z}] \in \mathbf{H}_n(K_{\epsilon_i}; G) : \epsilon_i \in \mathcal{L}(\mathbf{z})\}$ is a basis for $\mathbf{H}_n(K_{\epsilon_i}; G)$ for each i. Since $\mathcal{B}$ is a persistent homology cycle basis for $L'_\bullet$, it is a straightforward exercise in definition checking to verify that $\mathcal{D}$ is a persistent homology cycle basis for $L_\bullet$.

This establishes our primary objective:

THEOREM 2.8 (Correctness of Algorithm 2). *Algorithm 2 returns a bona-fide persistent homology cycle basis of $K_\bullet$.*

3 SUPPLEMENTARY TABLES AND FIGURES

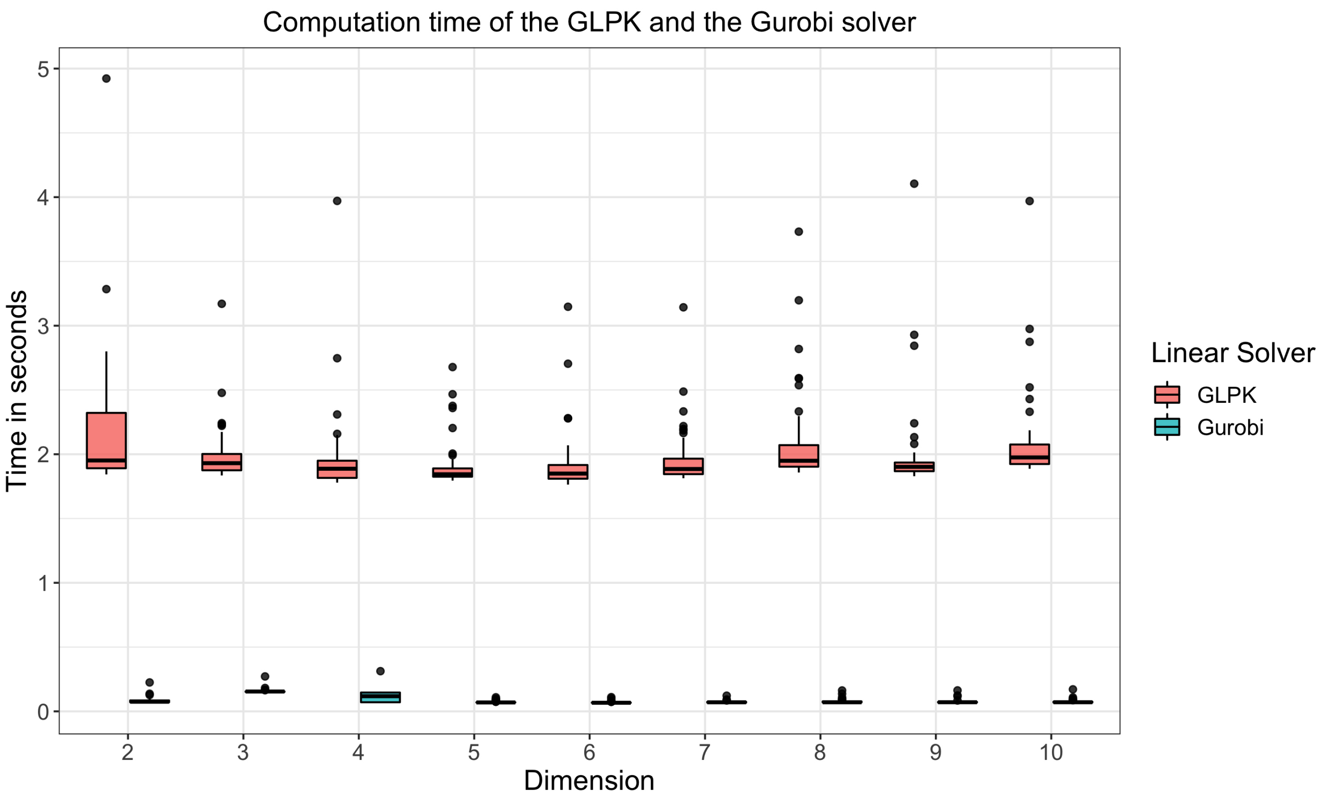

Figure S1: Computation time of the GLPK linear solver (red) and the Gurobi linear solver (green) to solve the uniform/length-weighted edge-loss minimal problems in Algorithm 1. We perform experiments on 90 data sets, 10 for each dimension 2-10, generated from the normal distribution. The horizontal axis is the dimension of the data set, and the vertical axis is the time it takes to solve an optimization problem. We observe that the Gurobi solver is consistently faster than the GLPK solver and that computation time seems fairly constant across dimension.

Table S1. Classifying the coefficients of the optimal cycles for all of the real-world data discussed in Section 5.1 as well as all of the synthetic sets discussed in Section 5.2. The rows are labeled by the coefficient type of the cycle representatives: “Integral” means the coefficients for the cycle representative $\mathbf{x}$ are in $\mathbb{Z}$ and “In $\{-1, 0, 1\}$” means the coefficients for the representative $\mathbf{x}$ are in $\{-1, 0, 1\}$. For the columns, $\mathbf{x}$ represents the optimal representative with its superscript indicating the type of optimization problem: I for integer programming and NI for linear programming, and its subscript indicating the type of optimal cycle: $E\text{-}Len, E\text{-}Unif, T\text{-}Unif$ refer to edge loss length-weighted minimal (minimizing total length of 1-simplices), edge loss uniform (minimizing total number of 1-simplices), and triangle loss uniform (minimizing the number of 2-simplices a cycle representative bounds), respectively.

**Edge-loss filtered homological optimal cycles** (Program (14))								
	**Randomly Generated Data Sets**				**Real-World Data Sets**			
**Coefficient Type**	$\mathbf{x}^{I}_{E\text{-}Len}$	$\mathbf{x}^{NI}_{E\text{-}Len}$	$\mathbf{x}^{I}_{T\text{-}Unif}$	$\mathbf{x}^{NI}_{E\text{-}Unif}$	$\mathbf{x}^{I}_{E\text{-}Len}$	$\mathbf{x}^{NI}_{E\text{-}Len}$	$\mathbf{x}^{I}_{E\text{-}Unif}$	$\mathbf{x}^{NI}_{E\text{-}Unif}$
**Integral**	100%	100%	100%	100%	100%	100%	100%	100%
**In** $\{-1, 0, 1\}$	100%	100%	100%	100%	100%	100%	100%	100%
**Edge-loss persistent homological optimal cycles** (Program (8))								
	**Randomly Generated Data Sets**				**Real-World Data Sets**			
**Coefficient Type**	$\mathbf{x}^{I}_{E\text{-}Len}$	$\mathbf{x}^{NI}_{E\text{-}Len}$	$\mathbf{x}^{I}_{T\text{-}Unif}$	$\mathbf{x}^{NI}_{E\text{-}Unif}$	$\mathbf{x}^{I}_{E\text{-}Len}$	$\mathbf{x}^{NI}_{E\text{-}Len}$	$\mathbf{x}^{I}_{E\text{-}Unif}$	$\mathbf{x}^{NI}_{E\text{-}Unif}$
**Integral**	100%	100%	100%	100%	100%	100%	100%	99.94%
**In** $\{-1, 0, 1\}$	100%	100%	100%	100%	100%	100%	100%	99.94%
**Triangle-loss optimal cycles** (Program (15))								
	**Randomly Generated Data Sets**				**Real-World Data Sets**			
**Coefficient Type**	$\mathbf{x}^{I}_{T\text{-}Unif}$	$\mathbf{x}^{NI}_{T\text{-}Unif}$	$\mathbf{x}^{I}_{T\text{-}Area}$	$\mathbf{x}^{NI}_{T\text{-}Area}$	$\mathbf{x}^{I}_{T\text{-}Unif}$	$\mathbf{x}^{NI}_{T\text{-}Unif}$	$\mathbf{x}^{I}_{T\text{-}Area}$	$\mathbf{x}^{NI}_{T\text{-}Area}$
**Integral**	100%	99.99%	100%	100%	100%	100%	100%	100%
**In** $\{-1, 0, 1\}$	100%	99.99%	100%	100%	100%	100%	100%	100%

REFERENCES

Cohen-Steiner D, Edelsbrunner H, Morozov D. Vines and vineyards by updating persistence in linear time. *Proceedings of the twenty-second annual symposium on Computational geometry* (2006), 119–126.

de Silva V, Morozov D, Vejdemo-Johansson M. Dualities in persistent (co)homology. *Inverse Problems* **27** (2011) 124003, 17. doi:10.1088/0266-5611/27/12/124003.

Henselman G, Ghrist R. Matroid Filtrations and Computational Persistent Homology. *ArXiv e-prints* (2016).

Henselman-Petrusek G. *Matroids and canonical forms: theory and applications*. Ph.D. thesis, University of Pennsylvania (2017).

Wu P, Chen C, Wang Y, Zhang S, Yuan C, Qian Z, et al. Optimal topological cycles and their application in cardiac trabeculae restoration. *Information Processing in Medical Imaging, IPMI* (2017), 80–92.

Edelsbrunner H, Harer J. Persistent homology—a survey. *Discrete & Computational Geometry - DCG* **453** (2008). doi:10.1090/conm/453/08802.

Obayashi I. Volume-optimal cycle: Tightest representative cycle of a generator in persistent homology. *SIAM Journal on Applied Algebra and Geometry* **2** (2018) 508–534.
